# Using Evidence-Based Pedagogical Approaches to Pivot from In-Person to Online Training in a D43 Program during the COVID-19 Pandemic

**DOI:** 10.4269/ajtmh.22-0150

**Published:** 2022-09-12

**Authors:** Erasto V. Mbugi, Claudia A. Hawkins, Lisa R. Hirschhorn, Sylvia Kaaya, Elizabeth N. Christian, Amani Anaeli, Emmanuel Balandya, Candida Moshiro, Denise L. Drane

**Affiliations:** ^1^ Muhimbili University of Health and Allied Sciences, Dar es Salaam, Tanzania;; ^2^ Robert J. Havey, MD Institute for Global Health, Northwestern University, Chicago, Illinois;; ^3^ Division of Infectious Diseases, Northwestern University, Chicago, Illinois;; ^4^ Department of Medical Social Sciences, Northwestern University, Chicago, Illinois;; ^5^ Searle Center for Advancing Learning and Teaching, Northwestern University, Chicago, Illinois

## Abstract

The COVID-19 pandemic caused significant disruption to medical education globally. Fogarty International Center (FIC) training programs, designed to strengthen research capacity in low- and middle-income countries (LMICs), through partnerships between United States and LMIC institutions were particularly vulnerable to COVID-19 disruptions. We adapted short-term training for our FIC HIV Patient-Centered Outcomes Research program in Tanzania to the virtual environment using synchronous, asynchronous, and blended approaches and a variety of teaching pedagogies. We evaluated the acceptability and effectiveness of the new trainings among trainees and facilitators using a mixed-methods approach. Ninety percent of trainees and Muhimbili University of Health and Allied Sciences (MUHAS) facilitators agreed that the virtual training methods used were effective. Trainees reported high levels of satisfaction with the technology, group work, and relevance to their research. More than 50% of trainees and MUHAS facilitators agreed that learning in the virtual environment was as effective as, or more effective than, traditional in-person learning. However, they desired more interaction, opportunities to ask U.S. facilitators questions, and choices about topics for online versus in-person trainings. Two-thirds of U.S. facilitators agreed that the virtual delivery method was an effective way for participants to learn the material, although they also rated interaction less favorably. Virtual training incorporating pedagogical best practices of blended learning and traditional teaching online was a feasible, acceptable, and effective way of conducting research training to junior scientists during COVID-19. Virtual learning could become an integral part of post-pandemic training with some adaptation to improve interactions.

## INTRODUCTION

Building research capacity in institutions in low- and middle-income countries (LMIC) countries is a priority to increase the relevance and uptake of evidence and research findings.^1^^,^^2^ Since 1968, the Fogarty International Center (FIC) of the NIH has had a mission of supporting and facilitating research conducted by investigators from these countries in partnership with U.S. research institutions, along with training the next generation of scientists to address global health needs.^3^ Since 1968, FIC has provided training support to more than 6,000 scientists from more than 100 countries.^4^

One grant mechanism funded through FIC is the D43 International Research Training Grant, which supports developing and strengthening scientific leadership and skills for research in LMIC institutions.^5^ The D43 program aims to strengthen research capacity in a defined HIV scientific area at a specific LMIC research institution with a collaborating U.S. partner. Programs typically support a broad range of short (< 3 months), medium (3–6 months), and long-term (> 6 months) training to build the capacity needed for trainees to pursue a career in health research in their home country. Short-term training includes skills-building workshops and short courses or mentoring that focus on a range of areas from research methodology to research ethics, program and grants administration, grant writing, more general scientific writing, and other relevant topics.^6^ These trainings are usually conducted at either the U.S. or LMIC institution and have traditionally occurred in person using lectures and active learning techniques and required international travel.

The COVID-19 pandemic caused significant disruptions to medical research training globally, including the FIC D43 programs, with substantial restrictions in travel to and from partner institutions for both Northwestern and Muhimbili University of Health and Allied Sciences (MUHAS) faculty and international trainees. These disruptions resulted in many training opportunities being postponed or canceled or quickly shifted from in person to online. These shifts to online training required several adaptations, including new formats and pedagogies to accommodate teaching and work virtually.^7^ To add to the challenge of continuing to build research capacity during the COVID-19 pandemic through different pedagogies, many trainees experienced increased clinical, administrative, and research responsibilities directly linked to COVID-19, which interfered with the D43 training and career progression for junior scientists more widely.^8^^,^^9^ In response, training formats had to be adapted to ensure the same quality and effectiveness of learning and be flexible enough to allow trainees to manage these new competing priorities.^10^^–^^13^

Researchers at MUHAS in Tanzania and Northwestern University in the United States collaborated in 2019 to establish the HIV Patient-Centered Outcomes Research (PCOR) program (D43TW010946) funded by the NIH FIC. The HIV PCOR program aims to build the capacity of Tanzanian researchers to conduct PCOR and improve the quality of healthcare services delivery for people living with HIV (PLWH). During the first wave of the COVID-19 pandemic in April 2020, the program was supporting two trainees enrolled in a master’s and PhD research program, respectively. Short-term trainings for larger groups of participants, including an introduction to PCOR and related skills-building, mentoring workshops, and responsible conduct of research had also been held. These trainings were conducted in person and facilitated collaboratively by faculty from the United States and Tanzania. After the onset of the pandemic, the Northwestern–MUHAS PCOR leadership worked to adapt and design short courses and other trainings, incorporating a variety of pedagogical approaches to continue meeting research training needs with the minimum possible interruption. The design process built on previous experiences of the team and the existing and emerging literature around best practices for virtual and blended learning.

This article describes how we adapted short-term trainings within our D43 program by including new formats and pedagogies. We also describe reactions of trainees and MUHAS and Northwestern facilitators to these changes and how COVID-19 impacted learning and teaching in the context of our D43 program. These results are relevant to the development of best practices for maintaining research capacity building through virtual and blended training during times of interruption, such as the COVID-19 pandemic. The study also has important implications for the design of international research training programs more broadly. Providing in-person training by faculty from the United States is expensive and time-consuming. If online and blended research training models can be shown to be effective and are acceptable to trainees and faculty, they could be used more widely as a core component of capacity building within D43 programs more generally, not just in times of emergency.

## METHODS

### Training before and during the COVID-19 pandemic.

In Table 1 the first year of the grant (2019), facilitators conducted two 3-day in-person workshops on PCOR. Both workshops included in-person facilitation by faculty from Northwestern and MUHAS. Pedagogy reflected adult learning, including small group work and practical applications of the skills during and after the workshops. Before the first COVID-19 wave, one PhD student attended classes as planned at Northwestern.

**Table 1:** Timeline and description of the format of and pedagogy used in training activities pre-COVID and during COVID

Title	Date	Format	Pedagogy
Pre-COVID: September 23, 2019–February 28, 2020			
Workshop: Orientation to PCOR	Sept. 23–25, 2019	In person	Lectures (Northwestern facilitators), small group work
Workshop: Mentorship	Feb. 24–26, 2020	In person	Lectures (Northwestern facilitators), small group work
COVID-19 restrictions introduced: March 1, 2020			
MOOC and small group: PCOR	Monthly starting July 2020 (alternating with virtual journal club)	Blended asynchronous and synchronous	Flipped classroom: students view unit of existing MOOC and complete prework Synchronous virtual sessions including review by trainee, discussion with Northwestern faculty facilitator, using Padlet questions
Workshop: PCOR and Patient-Reported Outcome Measures	Oct. 21–22, 2020	Blended asynchronous and synchronous	Prework asynchronous viewing prerecorded lecture Small-group work in person at MUHAS (trainees and MUHAS facilitators) in the morning Synchronous virtual zoom sessions in afternoons with Northwestern facilitator
Workshop: Scientific Writing	March 29–April 6, 2021	In person	Lectures (MUHAS facilitators), small group work
Workshop: Responsible Conduct of Research	November 10 and 13, 2020; March 8 and 10, 2021	In person	Face-to-face sessions Materials circulated for trainee self-reading 2 days before training
Workshop: Qualitative analysis	June 29, 2021 (Part 1); December 14–15, 2021 (Part 2)	Blended asynchronous and synchronous	Prework asynchronous viewing prerecorded lecture Small-group work in person at MUHAS (trainees and MUHAS facilitators) Synchronous virtual zoom sessions in afternoons with Northwestern facilitator
Journal Club	Monthly (alternating with MOOCs) starting April 2020	Virtual	Asynchronous prework: review journal articles and respond to questions Virtual synchronous with Northwestern facilitators and trainee cofacilitator: presentation (trainee) and facilitated discussion, Padlet questions (www.padlet.com)
Supplemental cross NU-D43 best practices in research	Monthly	Virtual	Virtual synchronous (zoom): lecture presentation by Northwestern facilitators and discussion

MOOC = massive open online course; MUHAS = Muhimbili University of Health and Allied Sciences; PCOR = Patient-Centered Outcomes Research. Blended indicates combined in person and virtual sessions.

Governments across the globe instituted pandemic-induced restrictions to travel after March 2020. Both MUHAS and Northwestern D43 grant leads agreed to implement virtual learning sessions or a combination of virtual with in-person learning formats at MUHAS to sustain ongoing PCOR training. Faculty at MUHAS ensured in-person trainings were equipped with appropriate safety precautions (masking, sanitizing, and social distancing). Support for Internet connectivity for trainees was strengthened to allow for expanded participation in the virtual offerings. We leveraged an existing massive open online course (MOOC) on PCOR to supplement the PCOR learning for our trainees. The newly adapted learning opportunities included an 8-month series of facilitated synchronous and asynchronous sessions cofacilitated by a Northwestern faculty and a MUHAS trainee. Before each monthly synchronous session, trainees viewed the preassigned MOOC session, completed a worksheet, and reviewed relevant supplementary articles. The role of the trainee co-facilitator was to summarize the MOOC sessions and cofacilitate the discussion as an opportunity to develop their teaching and presenting skills. Additional tools used during synchronous sessions included an online digital discussion board tool and a zoom chat function.

We supplemented the MOOC sessions by synchronous online journal clubs cofacilitated by a Northwestern faculty and MUHAS trainee focusing on PCOR articles relevant to Tanzania and the research focus of the D43. Trainees also participated in monthly synchronous online seminars offered through an Agency for Healthcare Research and Quality funded K12 research training program in PCOR at Northwestern (A Chicago Center of Excellence in Learning Health Systems Research Training; ACCELERAT). In addition, a monthly online lecture series on best research practices was established and conducted with three partner D43 training programs at Northwestern to build capacity for areas including project management and budgeting. Finally, we conducted four 2-day workshops with synchronous and asynchronous components on Patient-reported Outcome Measures (PROMS), Patient Reported Experience Measures (PREMs), and qualitative methods. These workshops were cofacilitated by Northwestern and MUHAS facilitators. Facilitators and cofacilitators used a blended approach combining asynchronous sessions, where trainees previewed prerecorded lectures and read supplemental course material, with small group work either in-person or virtually as well as synchronous online classes and discussion sessions. Faculty at MUHAS also conducted one in person manuscript writing workshop between two COVID-19 waves and two in-person responsible conduct in responsible conduct of research workshops.

### Evaluation methods.

We conducted a mixed-methods evaluation using online surveys and an online focus group. Broad aims of this evaluation were to 1) examine the reaction of trainees, MUHAS, and Northwestern facilitators to the adapted pedagogy; 2) examine trainee and MUHAS and Northwestern facilitator perceptions of the effectiveness of the adapted pedagogy; and 3) identify strengths and weaknesses of the adapted pedagogy. Surveys consisted of quantitative Likert scale rating questions and open-ended questions. Survey and focus group data were triangulated.

#### Trainee and Tanzanian facilitator reaction to the training.

In June 2021, facilitators and trainees were invited to complete a survey about their experience with the adapted pedagogy during COVID-19. The survey focused on the effectiveness of the small-group sessions in enhancing learning, suitability of the learning environment, quality of asynchronous sessions, length and timing of the training, how well the technology worked, and relevance of the training content to trainees’ research.

Respondents were also asked to rate the effectiveness of aspects of online learning compared with in-person learning. The components of the online workshops that were rated included learning resources, the interaction between facilitators and participants, participant participation and attendance, learning of the material, and how learning was assessed. Facilitators were asked how prepared they felt to teach the material virtually and also asked open-ended questions about which aspects of the technology worked and which did not. When appropriate, questions were asked using a 5-point Likert scale. Finally, all respondents were asked if they would like to continue participating in virtual workshops in the future, the types of training they would prefer to be conducted virtually, and which types of training they would prefer to be conducted in person.

#### Qualitative.

Seven of the trainees enrolled in formal training programs (PhD, postdoctoral, master’s) participated in a 1-hour online focus group facilitated by the program evaluator (Denise Drane) in June 2021. The semistructured focus group protocol included questions about the different training modalities (MOOC, online journal club, and post-COVID PROMS workshop). Questions about the MOOC focused on effectiveness in helping trainees learn about PCOR, the most important aspects of the content and format, and the helpfulness of the supplementary monthly MOOC virtual sessions. Questions about the online journal club focused on how effective it was in helping trainees to learn how to conduct PCOR, experiences participating in the online journal club compared with participating in an in-person journal club, and suggestions for improvement. Questions about the PROMS workshop focused on trainee experiences and how the experience of online learning compared with in-person learning. Finally, trainees were asked about the challenges associated with online learning, how they addressed these challenges, and what aspects of online programming they would like to continue after COVID, either in their present form or a modified form.

#### United States–based Northwestern University facilitator reactions and perceptions of the training.

Northwestern facilitators completed a brief survey that asked about their perspectives on the training. The survey questions included the same Likert scale questions about experience, preparation, and aspects of the technology that worked and did not work as the survey described earlier. The survey contained some additional questions about whether the U.S.-based facilitators would like to continue facilitating virtual workshops in the future, what topics and learning formats work effectively virtually, and which ones should be conducted in person.

### Data analysis.

Quantitative data from the survey questions were analyzed using descriptive statistics (frequencies). The evaluator extracted themes from the open-ended surveys and focus group responses using a constant comparative approach.^14^

## RESULTS

### Trainee and faculty facilitator (Tanzania) experience of the training.

**Figure 1.**
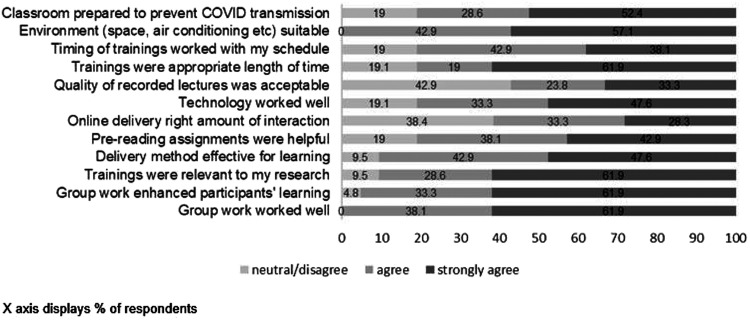
Trainee and Tanzanian facilitator ratings of their experience of the virtual trainings.

Evaluators sent the survey to 76 trainees and facilitators from Tanzania; 23 (30.3%) responded (Figure 1). These included 13 training participants (including seven in the formal degree or postdoctoral training sessions supported by the D43) and 10 facilitators who participated in the training.

Overall reaction to the trainings was positive. All trainees and facilitators agreed that the trainings were relevant to their research, that the group work worked well, and that prereading assignments were helpful. Ninety percent agreed that the virtual training approach or delivery method was effective. Only 14 (19%) trainees had concerns about the quality of recorded lectures and technology in general. Fifteen percent disagreed that the classroom preparation was favorable to prevent COVID-19 transmission. A third disagreed that online delivery had the right level of interaction and involvement.

### Trainee and faculty facilitators’ (Tanzania) perceptions of the effectiveness of virtual and in-person training.

**Figure 2.**
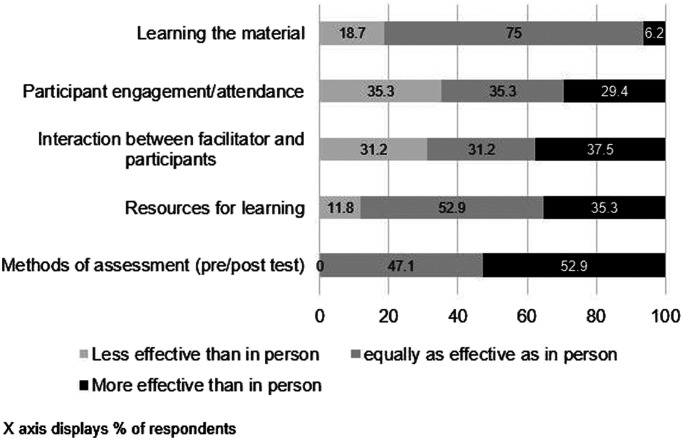
Training participants’ ratings of the effective of the online and virtual blended learning relative to the in-person workshops and lectures

Over half of the survey respondents rated the virtual methods of assessment, resources for learning, and ability to learn the material either “as effective” or “more effective” than training conducted in-person (Figure 2). However, a third thought the level of interaction and participant engagement was less effective in the virtual training environment than in-person.

### Focus group discussions with trainees.

All seven trainees supported by the HIV PCOR D43 through PhD, master’s, or postdoctoral programs expressed positive views about the virtual training. All agreed that the virtual training was valuable. They appreciated the flexibility of the asynchronous learning sessions and the ability to replay lectures to assist their learning of challenging material. They particularly appreciated the discussions with experts and found them helpful. Having the opportunity to ask questions was important to the trainees. Overall, they said that they preferred in-person learning because they feel that it is easier for them to ask questions and that there was more interaction.

Trainees agreed that the virtual training sessions should continue but with some modifications, including the following:

- more time for discussion (i.e., longer discussion sessions or additional discussion sessions);
- allowing trainees more choice in the topics included in the virtual learning;
- allowing trainees to choose which topics are online and which topics are in-person (e.g., they felt that complex material such as statistics and psychometrics were best done in person);
- when trainees are facilitating or co-facilitating, have two trainees facilitate sessions so that there is not as much burden on one person; and
- provide trainees with the opportunity to contact Northwestern facilitators by e-mail to ask follow-up questions.

Finally, the trainees suggested that it would be helpful for them to debrief with the program directors after the end of virtual training units to talk about gaps in their learning, areas that they still need to learn more about, and how they can address these gaps and needs in the future.

### Northwestern facilitator experience of the training.

**Figure 3.**
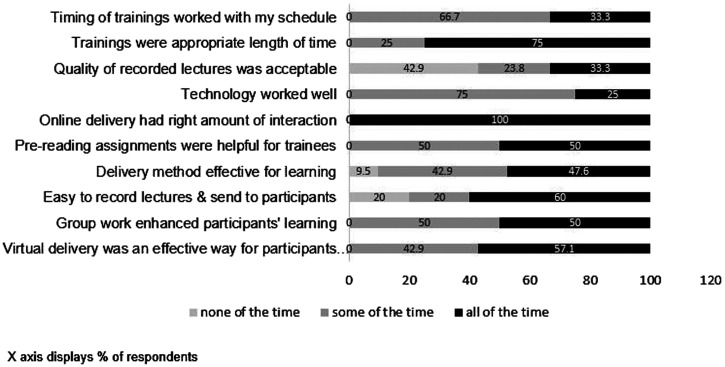
U.S. facilitator experience of the virtual trainings.

All eight Northwestern faculty facilitators who participated in the MOOCs or workshops responded to the survey about their experience (Figure 3). Seventy-five percent agreed that the virtual training was an appropriate length of time, although only 30% responded that the timing worked well with their schedule all the time. Fifty to sixty percent felt that the virtual training delivery method, ease of recording, prereading, and group assignments were effective and helpful. Only 25% indicated that technologies worked well all the time. All respondents felt that online training delivery had the right level of interaction and involvement only some of the time. Respondents agreed that much of the learning (viewing modules, lectures, etc.) should occur in trainees’ own time, responding to an open-ended question about which topics and formats were best virtually and best in person. In doing so, this would allow time for more in-depth discussions of subject material during synchronous virtual sessions. Some facilitators felt that it would be more appropriate to have participants attend synchronized sessions in their own virtual space rather than in a room together with other trainees, so they felt more comfortable asking questions. Facilitators agreed to find additional novel ways to improve trainee–facilitator interactions.

## DISCUSSION

In this study, we found that virtual training incorporating pedagogical best practices of blended learning and traditional teaching online was a feasible, acceptable, and effective way of conducting research training to junior scientists during COVID-19 across two institutions on two continents. We demonstrated the ability to switch rapidly and seamlessly between planned in person to virtual platforms in response to COVID-19 restrictions and implemented technologies and resources that will strengthen the capacity-building activities beyond the COVID-19 pandemic. We also identified additional areas where capacity building is needed, both from Northwestern and MUHAS facilitators in terms of technology and in small group facilitation.

The COVID-19 pandemic has forced educators worldwide to adopt online or virtual pedagogy rapidly to teach students in various educational settings, including those involved in training young researchers.^15^ Migration to virtual platforms offers many opportunities to introduce a range of interactive methodologies for learning, including abbreviated lectures, video lectures for asynchronous viewing, small group work, discussion groups, case studies, forums, and natural environments for promoting collaborative learning. Virtual training offers several benefits, including improved equity and reaching more extensive and diverse audiences.^16^ It has also had many individual benefits to the learner, including opportunities for students to personalize, extend, push each other to examine and re-examine academic ideas and content while improving their questioning, learning, and skills in presenting.^17^

During COVID-19, we successfully conducted a range of virtual training that were either a hybrid of in person and online (workshops, MOOCs) or led entirely online (supplemental lectures and journal clubs). The blended learning approaches where trainees had access to online educational material before the training followed by synchronous sessions with facilitators during the training were well received by trainees and facilitators from both the United States and Tanzania. This combination of asynchronous and synchronous approaches to facilitate remote learning is highly effective in other settings.^18^ Having access to pretraining videos and readings during the workshops allowed trainees to learn at their own pace with greater flexibility and facilitators to explore topics in greater depth during the online synchronous sessions. In-person group work, incorporated into the workshops, allowed trainees to interact with peers and work together on assigned questions with the involvement of MUHAS cofacilitators with expertise in the subject material. All trainees and facilitators either agreed or highly agreed the in-person group work activities worked well. However, it is notable that even though the trainees were satisfied with the group work, in the focus group, they expressed a desire for even more interaction, including more time for discussion and more time to interact with facilitators (via e-mail) after sessions.

Technology provides the mechanism for implementing best pedagogies and learning resources and therefore is critical for successful online learning.^19^ Inadequate access to technologies, poor computer literacy, and unreliable systems all pose significant challenges to training online, particularly in LMIC.^20^ Overall, there were few concerns about technological capacity among trainees and facilitators in our program; 80% to 85% of trainees agreed that the technology used worked well and that the quality of recorded lectures was acceptable. However, only 25% of Northwestern facilitators agreed that technology worked well all the time. This difference in trainee and Northwestern facilitators’ views may have reflected differing expectations regarding the technology or more significant challenges delivering or recording lectures versus viewing. Notably, 20% of Northwestern facilitators involved in the MOOCs and workshops mentioned that it was “easy to do pre-recordings none of the time,” reflecting an area where support is needed.

A significant advantage of moving our trainings virtually was bringing in external experts to the training at a minimal cost. Our blended series in PCOR methodology combined a series of MOOC modules completed independently by trainees with monthly synchronous sessions with a facilitator, using five PCOR experts from Northwestern who would have otherwise not been able to travel to Tanzania because of high costs and logistics given the short interaction. Although virtual training generally allows for more flexibility with scheduling, time zone differences are an essential consideration when working internationally; only a third of Northwestern facilitators in our survey agreed that the training timing worked well with their schedules.

Another advantage of using this blended approach was offering training to a larger and more diverse group of trainees who may have been unaware of them or not able to attend in person. Often highly specialized research training offered through training grants is only available to trainees affiliated with them. For the first time since the grant’s inception, we included trainees from other D43s at MUHAS, many based in different institutions in Tanzania. This inclusion led us to develop a Tanzanian D43 training network through which we are currently exploring ideas for sharing access to training and other educational resources. The COVID-19 pandemic and rapid growth of remote learning have led to several intra- and interinstitutional initiatives like this in different settings.^21^

The move to online training also has potential benefits for equity in training programs. Inequities in partnerships between high income country (HIC) and LMIC institutions have been identified regarding the control and flow of resources and the setting of research and training agendas.^22^^–^^24^ Rabin et al.^25^ further noted that “these structural inequities, coupled with socially ingrained attitudes that equate power with knowledge, reinforce the perception that individuals from HIC institutions are positioned best to play the role of the teacher.” MUHAS faculty and trainees were more involved in the virtual training than in the in-person training. In the virtual training where trainees served as facilitators and peer-facilitators in flipped classrooms and blended sessions related to the MOOCs, they elevated their role as teachers. This teaching role may help transform perceptions about the capacity of LMIC faculty as teachers and who is best positioned to play the role of teacher.

Finally, implementing virtual training at a rapid pace has significantly improved the capacity of MUHAS to accommodate and sustain virtual trainings in the future. Trainees at MUHAS also now have access to a broader pool of expert facilitators and mentors and technological resources that would otherwise have not been available. Ninety percent of our trainees were favorable to maintaining virtual training online. However, some focus group trainees thought some training might be better suited for virtual platforms than others. From a practical perspective, training activities conducted over more extended periods, like our blended MOOCs model or training, can serve a more extensive and more diverse pool of trainees without compromising on quality; they may be better suited for online or blended sessions and small in-person group work to enhance learning from synchronous and asynchronous virtual sessions.

Student’s learning has also been shown to be greatly enhanced through engagement with others.^26^ One of the main concerns expressed during our virtual and blended trainings was about the level of student–student and student–facilitator interaction and communication. Student interactions have been identified as one of the most significant challenges in distance learning.^27^ Although facilitators and trainees rated many aspects of the virtual trainings satisfactorily, both groups responded less favorably about the opportunities for interaction during virtual trainings and had concerns about levels of engagement. No Northwestern facilitators agreed that “online delivery had the right level of interaction and involvement” all the time; a third of MUHAS facilitators and trainees disagreed with or were neutral about this statement. A third of the trainees said that interaction and engagement/attendance were less effective than in-person training. In focus group discussions, trainees specifically mentioned that opportunities to interact with facilitators and ask questions during and after the sessions was limited. The format of one of the workshops where all participants were in the same room during an online synched session may have made it more awkward for participants to ask facilitators questions during the training. Additional opportunities such as office hours may address some of these challenges, which we implemented in our most recent qualitative research training.

Several strategies effectively improve peer–peer and student–facilitator interaction and engagement during virtual training, including embedding synchronous communications into videos such as polls during teaching audiences^28^ or using other technologies to support active learning (e.g., padlet, use of chat, gamification).^29^ Although effective, many technologies and pedagogies used to promote and sustain optimal interactions, particularly in the virtual space, require a significant amount of planning by facilitators.^30^ These additional efforts should be considered when offering compensation to facilitators and may also increase the cost of training. Creating breakout groups during online synchronous sessions or integrating sessions for in-person group work where trainees can connect through joint activities are recommended as best practices in online course design to improve communication and interaction.^31^ Finally, offering online office hours beyond the “classroom” for synchronous communication with facilitators, as we did for our qualitative research training in late 2021, and creating online discussion forums to sustain these connections is crucial for maintaining dialogue and getting expert feedback.

There are some limitations to this study. Our overall response rate was low among trainees due to some challenges completing the survey online. Although not part of the evaluation in this work, overall participation in trainings was notably lower than in prior in-person trainings. There could have been several reasons for this, including demands on time due to COVID-19 from work and family responsibilities, limitations with technologies, or lack of incentives. An additional limitation of this study is that we could not do meaningful comparisons of data from trainees and facilitators because of the relatively small numbers in each group. There were some trends that would be worthwhile examining by combining data from the present study with data from future cohorts of trainees and facilitators. For example, trainees had lower rates of agreement with the statement that “the online training had the right amount of interaction” than the MUHAS and Northwestern facilitators. Finally, although the virtual trainings were highly regarded by facilitators and trainees, we did not measure and compare actual learning in virtual and in-person trainings which would have been helpful to determine whether the virtual trainings adequately addressed knowledge gaps, the transfer of knowledge differed between training formats, and what could be improved upon in the future.

In conclusion, we have demonstrated that expanding trainings to include or be wholly virtual while incorporating pedagogical best practices of blended learning and traditional teaching online are a feasible, acceptable, and effective way of conducting research training to junior scientists during COVID-19. Areas for capacity building need to be included during planning and implementation, whether in technology, virtual teaching, and facilitation in blended learning settings. Implementing strategies that encourage trainee–facilitator and trainee–trainee interaction during and after the trainings are an important consideration and create the most effective learning environment for trainees. The virtual platform could become an integral part of post-pandemic training with some adaptation.
